# Genomic Analysis of the SUMO-Conjugating Enzyme and Genes under Abiotic Stress in Potato (*Solanum tuberosum* L.)

**DOI:** 10.1155/2020/9703638

**Published:** 2020-06-24

**Authors:** Shantwana Ghimire, Xun Tang, Ning Zhang, Weigang Liu, Xuehong Qi, Xue Fu, Huaijun Si

**Affiliations:** ^1^College of Agronomy, Gansu Agricultural University, Lanzhou 730070, China; ^2^Gansu Provincial Key Laboratory of Aridland Crop Science, Gansu Agricultural University, Lanzhou 730070, China; ^3^College of Life Science and Technology, Gansu Agricultural University, Lanzhou 730070, China

## Abstract

SUMO-conjugating enzymes (SCE) and SUMO (Small Ubiquitin-Like Modifiers) genes are important components of SUMOylation. SCE has a crucial role during the SUMOylation process which acts as a catalyst to transfer SUMO to the target protein. Comprehensive studies on SCE and SUMO have been performed in some plants, but studies on these genes remain limited in potato. This study is aimed at exploring the role of *StSCE* and *StSUMO* genes in abiotic stress conditions. Nine and seven putative *StSCE*s and *StSUMO* genes, respectively, were identified using different methods and databases available for potato. Chromosomal localization showed that *SCE* and *StSUMO* genes are unevenly distributed on 7 different chromosomes. Potato genome database was accessed for the expression profile of *StSCE* and *StSUMO* genes, and these genes were differentially expressed in different tissues and organs during different phases of plant growth. The expression patterns on different treatments were further evaluated using qRT-PCR for all the *StSCE* and St*SUMO* genes. The expression was upregulated in *StSCE1/5/6* and *7* under salt and PEG treatment. *StSUMO 1/2* and *4* were upregulated under salt stress whereas *StSCE9* and *StSUMO2* and *4* were observed downregulated under PEG treatment. The results of this study could be useful to explore the role of *StSCE* genes in potato improvement.

## 1. Introduction

Posttranslational modifications (PTMs) of proteins are the crucial process for the regulation of normal functioning of biological activities and play a crucial role during various stress conditions [1–3]. PTM is involved in different molecular processes, cellular signaling, and different developmental stages by modifying specific lysine residue in protein substrate covalently or interacting with proteins noncovalently [4–8]. The biochemical steps of SUMOylation are similar to those of ubiquitination [9], but SUMO does not tag protein for degradation [7] and comprises three enzymes: SUMO-activating enzyme (E1, SAE1/SAE2), SUMO-conjugating enzyme (E2, SCE), and SUMO ligase (E3) [1, 10]. SUMO is a family of a polypeptide with around 110 amino acid tags which are distantly related to Ub (ubiquitin) [11–13]. SAE is a heterodimer that acts as a catalyst to activate SUMOylation. The SCE conjugates the SUMO carboxyl terminus glycine to lysine Ɛ-amino group in the substrate either alone or with the help of E3 [14] thereby resulting modifications in the stability of proteins, interaction between proteins, nuclear transport, localization of proteins, and interaction between proteins and DNA [15]. The number of SUMO proteins identified to date contains an acceptor Lys within a consensus motif *ψ*KX (D/E) (*ψ* is a large hydrophobic residue) and plays an important role to stabilize the interaction between the E2 enzymes and its specific substrates [16–19]. Despite the fact that SCE has a distinct capacity to recognize its specific substrate even in the absence of E3 ligase, limited information is found in *SCEs* in plants. The studies done so far have suggested that single gene encodes SCE and several genes encode UbE2 (Ubiquitin-Conjugating Enzyme) in yeast and humans [1].

Potato (*Solanum tuberosum* L.) is globally considered as the third most important nongrain crop which is grown for human consumption, and total yield is more than 300 million metric tons worldwide [20] and cultivated worldwide in nearly 125 countries food securing one billion population [21]. Potato is considered as an important food crop all over the world but highly affected by drought stress because of its shallow root system [22, 23]. However, potato production is severely influenced by abiotic stresses [24]. There are insufficient studies on the mechanism of *SUMO* and *SCE* in response to different stresses in crops. All of the recent studies on potato [25, 26] focused on SUMO E3 ligase SIZ1, and functional characterization SCE in monocot plant, rice, has been published [8]. This research will explore the roles and functions of *StSCE* and *StSUMO* genes during abiotic stress conditions which could be used to explore their roles in potato.

## 2. Materials and Methods

### 2.1. Identification and Phylogenetic Analysis

For exploring the SUMOs and SCEs in potatoes, BLAST searches were performed in the potato genome database PGSC (http://solanaceae.plantbiology.msu.edu/pgsc_download.shtml) using *Arabidopsis* and rice SUMO and SCE gene sequences. HMM (Hidden Markov Models) was used to search the candidate potato SUMO and SCE proteins as a query. The potato protein sequences were downloaded from Phytozome 12.1(https://phytozome.jgi.doe.gov/pz/portal.html#), and SCE (PF00179) and SUMO-conserved domain sequences (PF11976) were obtained from Pfam 31.0 database (http://pfam.xfam.org/). The putative members' exploration of SUMO and SCE in potato was done by the combined search of HMMER of hmmbuild and hmmsearch program with default parameters. The UQ-con domain of all the SCE candidates and Rad60-SLD domains of SUMO gene candidates were confirmed by using Interpro (http://www.ebi.ac.uk/interpro/), SMART (http://smart.embl-heidelberg.de/) and HMMER (https://www.ebi.ac.uk/Tools/hmmer/)online tools.

A phylogenetic tree was constructed to explore the evolutionary relationship among UQ-con domain proteins (SCE) and SUMO domain (Rad60-SLD) in various plant species. *Arabidopsis*, rice, and potato SCE and SUMO protein sequences were used to create a phylogenetic tree using MEGA 7.0 [27]. Multiple sequence alignments with default parameters were done by using MUSCLE [28]. The neighbor-joining method with 1000 bootstrap replicates was used to construct a phylogenetic tree.

### 2.2. Plant Materials

The potato cultivar “Atlantic” (USDA pedigree no. B6987-56) [29] used in this study was obtained from the Gansu Provincial Key Laboratory of Arid land Crop Science, Lanzhou. The in vitro plantlets were cultured in Murashige and Skoog [30] liquid medium with 3% sucrose and pH 5.8. The plantlets were kept in the growth chamber with 16 h light/8 h dark photoperiod and temperature of 23 ± 1°C. After 4 weeks, the MS liquid medium was replaced with 150 mM NaCl for salt stress and 20% PEG 6000 for drought stress. The whole plant without treatment was collected as a control sample, and other samples were collected after 4, 8, 12, and 16 h of treatments. The collected samples were rapidly frozen in liquid nitrogen and stored at -80°C until further analysis.

### 2.3. qRT-PCR Analysis

NCBI Primer BLAST was used to design specific primers (Supplementary Table 1). The RNAsimple Total RNA Kit ((DP419) TIANGEN, China) was used for RNA extraction. The extracted RNA was treated using DNAase to get rid of genomic DNA in the sample. First-strand cDNA was synthesized by using the FastKing RT Kit (TIANGEN, China). SuperReal PreMix Plus (SYBR Green) was used for the quantitative real-time PCR (qRT-PCR) reaction following the manufacturer's instructions. The potato EF1*α* (PGSC0003DMG400023270) was used as an internal reference gene [31]. Lifecycler®96 was used to perform qRT-PCR analysis with three steps; preincubation was set to temperature 95°C for 900 seconds, and amplification was performed in two steps with 40 cycles. For the first step, the temperature was set to 95°C for 10 seconds, and during the second step, the temperature was maintained at 60°C for 20 seconds. Finally, the melting temperature was set to 95°C, 65°C, and 95°C for 15, 60, and 15 seconds, respectively. 2^(−*ΔΔ*Ct)^ was used to access the relative expression of genes [32]. The significant test was done by using R i386 3.6.1 software.

### 2.4. Gene Structure, Gene Duplication, Motif, and Chromosomal Localization

Exon/intron organization of the gene was identified by GSDS 2.0 (The Gene Structure Display Server tool (http://gsds.cbi.pku.edu.cn/)). ExPASy was used to calculate the biochemical characteristics of genes [33]. Gene duplication was analyzed and verified by using PGDD (http://chibba.agtec.uga.edu/duplication/index/locus), and the Ka/Ks ratio was calculated by using KaKs_Calculator 2.0. The MEME tool (http://meme-suite.org/tools/meme) [34] was used to predict the motifs with parameters of 10 limited number of motifs and others as default. MapInspect software was used for the chromosomal localization.

### 2.5. Analysis of *Cis-Acting* Elements, Protein Interaction, and SUMOylation Site

The sequences of *StSCE* and *StSUMO* genes were downloaded from the PGSC database and submitted to Plant CARE (http://bioinformatics.psb.ugent.be/ webtools/plantcare/html/)[35]. All the *cis-acting* elements of the promoter sequence were obtained and analyzed. All the protein sequences of the identified genes were submitted to STRING version 11.0, and interaction between the proteins was predicted [36]. GPS-SUMO was used to identify the SUMOylation sites and interaction motifs [37].

### 2.6. Expression Profile Analysis of Genes

RNA-seq data was accessed from the PGSC database to study and analyze the expression profile of *StSCE* and *StSUMO* genes [38]. Data on different stages of development, tissues, and organs and stress treatments were considered to analyze and visualized through a heat map by using the HemI 1.0: Heatmap Illustrator [39].

## 3. Results and Discussion

### 3.1. Identification of *StSCE* Genes, SUMOylation Sites, and SUMO-Interaction Motifs (SIMs) and Protein-Protein Interaction

Nine *StSCE* and seven *StSUMO* genes were confirmed in potato. *Arabidopsis* encodes a single SCE gene (At3g57870) [16] and eight SUMO genes. Similarly, three *OsSCE* and *OsSUMO* genes were present in rice [1, 8]. The information of the confirmed genes with their IDs is summarized in Supplementary Table 2. An isoelectric point and MW in SCE genes varied from 5.4 to 9 and 15715.3 to 18662.2, respectively. *StSCE1* was neutral whereas five *StSCE* were acidic and three were basic. Among 3 *OsSCE* genes, two were basic and one was acidic [8]. The isoelectric point of *AtSCE1* was 8.33 with molecular weight 1798.53. Similarly, isoelectric point ranged from 4.81 to 9.41 and MW ranged from 6797.66 to 12072.43. The MW of *OsSCE* genes varied from 15717.88 to 18045.58 which were almost similar to that of *StSCE* genes. *StSUMO1/5* and *6* were predicted to be slightly stable as their instability index ranged from 30 to 40 whereas remaining genes were predicted to be unstable having instability index more than 40, where 95.94 was the highest value. The GRAVY values for SCE genes were negative because of the hydrophilic proteins. Out of nine *StSCE* genes, five genes were in the nucleus and four in both cytoplasm and nucleus and they might be involved in regulating the activity of transcription factors thereby rapidly regulating cell metabolism. Likewise, in *StSUMO* genes, six were localized in the nucleus and one in the mitochondria, where *StSUMO3* was localized in both cytoplasm and nucleus. It suggested that *StSCE* and *StSUMO* genes are specific to the organelles which have specific functions in different environmental conditions. Recent findings reported that *OsSCE1*, *2*, and *3* were localized in the nucleus and slightly in the cytosol, while *OsSUMO1* was localized cytoplasm [8]. SUMOylation status of proteins involved in the stress response varies according to their localization in various organelles [10]. The identification of the SUMOylation site showed different consensus and nonconsensus sites of SUMOylation and SIMs [37]. SUMO-interaction motifs were present in all the genes except *StSUMO7*. In total, 134 SUMOylation sites were discovered, where each *StSCE1/3* and *4* contained one SUMOylation consensus site and the remaining others were SUMOylation nonconcensus sites. SUMOylation sites ranged from 3 to 13, where *StSUMO5* and *6* possessed the highest number of SUMOylation sites and *StSUMO7* possessed the lowest SUMOylation sites. Similarly, *StSCE1* and *5*, *StSCE3*, *StSCE4*, *StSCE 2* and *8*, *StSUMO2*, *StSUMO3* and *4*, *StSUMO1*, *StSCE9*, and *StSCE6* and *7* contained 12, 11, 10, 8, 7, 6, and 5 SUMOylation sites, respectively. Regarding the SUMO-interaction motifs (SIMs), all the *StSUMO* genes except *StSUMO7* contained one SIM whereas in *StSCE* genes the number of SIMs ranged from 3 to 6. *StSCE2* possessed the highest number of SIMS. *StSCE3/6/7/8* and *9* and *StSCE4* and *5* contained 4 and 3 SIMs, respectively. For details, please refer to Supplementary Table 3.

Three *StSCE* showed interaction with PGSC0003DMT400078207, PGSC0003DMT400079486 (ubiquitin-activating enzyme) *StSUMO1*, *StSUMO2*, *StSUMO3*, *StSUMO4*, *StSUMO5*, and *StSUMO6*, and the other six *StSCE* genes showed interaction with PGSC0003DMT400078207, PGSC0003DMT400079486 (ubiquitin-activating enzyme), *StSUMO1*, *StSUMO2*, and *StSUMO3*. For details, please refer to Supplementary Table 3. Similarly, all the *StSUMO* genes except *StSUMO7* showed interaction with different proteins. *StSUMO1/2* and *3* showed interaction with PGSC0003DMT400078207, PGSC0003DMT400079486 (ubiquitin-activating enzyme), and all the members of *StSCE*. These three *StSUMO* genes showed interaction with each other.

**Figure 1:**
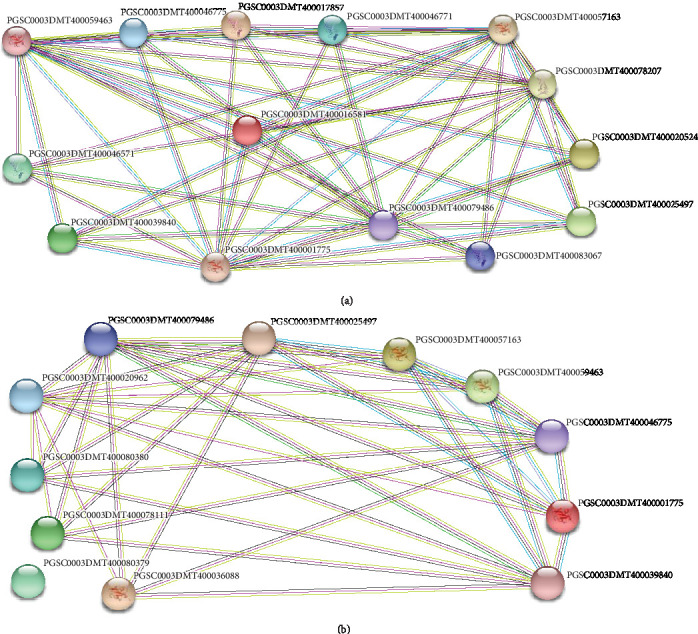
(a) Protein interaction between *StSCE* genes and (b) Protein interaction between *StSUMO* genes.

*StSUMO4/5* and *6* showed interaction with *StSCE2*, *StSCE3*, *StSCE5*, PGSC0003DMT400079486 (ubiquitin-activating enzyme), and PGSC0003DMT400020962 (SUMO ligase) (Figures 1(a) and 1(b)).

Some of these interacting proteins are the members of the SUMOylation pathway, such as SUMO, SUMO-activating enzymes, and ligases, and others are SUMO-modified target proteins which include the enzymes involved in DNA replication and inevitably affect DNA replication and cell proliferation.

### 3.2. Chromosomal Localization and Phylogenetic Analysis

**Figure 2:**
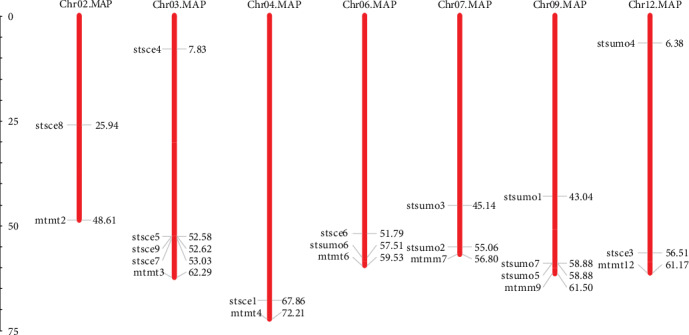
Distribution of *StSCE* and *StSUMO* genes in potato chromosomes. The map was constricted by using MapInspect.

Eight SCE genes in potato were distributed unevenly in five chromosomes. Seven *StSUMO* genes were found to be distributed in four different chromosomes (Figure 2). One of the SCEs in the potato which we renamed as *StSCE2* gene could not be traced in any chromosome. This gene might be located in the mitochondria or chloroplast, yet this needs further exploration. Four genes were localized in chromosome 3, and *StSCE8*, *StSCE1*, *StSCE6*, and *StSCE3* were mapped in chromosome 2, 4, 6, and 12, respectively. Most of the members of *StSCE* were clustered on the bottom of the chromosome except *StSCE4*. In *StSUMO* genes, *StSUMO6* was mapped in chromosome 6, *StSUMO2* and *3* in 7, *StSUMO1/5* and *7* in 9, and *StSUMO4* in 12 chromosomes, respectively. Likewise *StSCE* genes, most of the members of *StSUMO* were clustered on the bottom of the chromosome except *StSUMO4.*

**Figure 3:**
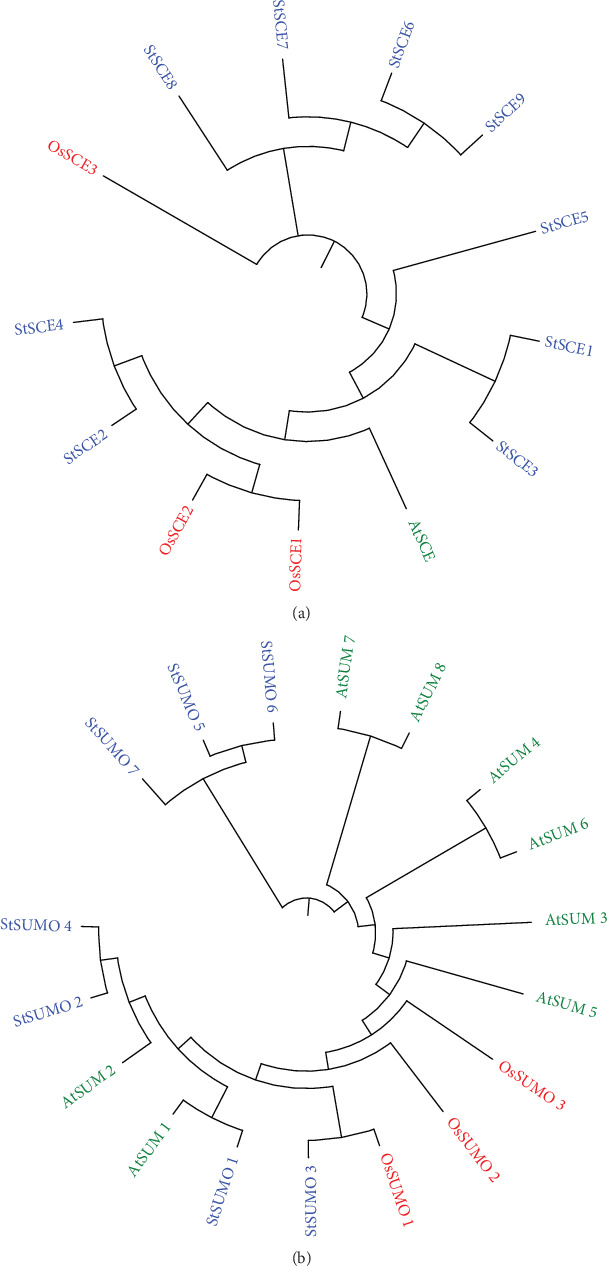
The phylogenetic tree of full-length SCE (a) and SUMO (b) proteins in *Arabidopsis*, rice, and potato. The evolutionary history was inferred using the neighbor-Joining method using MEGA7. The bootstrap consensus tree inferred from 1000 replicates.

For the better understanding of the phylogenetic relationship among *StSCE*s and *StSUMO*s in the plants and accessing the evolutionary history of these proteins, we performed phylogenetic analysis using multiple sequence alignment using a full-length sequence of 1 *Arabidopsis*, 3 rice, and 9 potato SCE genes and 8 *Arabidopsis*, 3 rice, and 7 potato SUMO genes (Figures 3(a) and 3(b)). The analysis showed that *StSCE2* and *StSCE4* share the same root with OsSCE1 and OsSCE2, so share the similarity on evolution with rice. *OsSUMO1* shares similarity with *StSUMO3*, and *AtSUM1* shares similarity with *StSUMO1*.

### 3.3. Analysis of the Gene Duplication

Analysis of gene duplication confirmed only one *StSCE* has segmental duplication, i.e., *StSCE1* with *StSCE3* where *E* value was *E* v8.00*E*-83 and the Ka/Ks ratio was 0.1069368. *StSCE1* and *StSCE3* were observed to have a very high homology during sequence alignment. So the segmental duplication might have taken place during the evolutionary process, and as the Ka/Ks ratio is less than 1, so *StSCE* genes were purified selection for evolution. Analysis of *Arabidopsis* and potato showed segmental duplication of AT5G62540 and AT1G14400 with *StSCE6* where respective *E* values were 1.00*E*-99 and 1.00*E*-110 with respective Ka/Ks value 0 and 0.0192164. As the *StSCE* in potato shoed similarity with that in *Arabidopsis*, it might suggest that these genes were conservative in the evolution and expansion and no gene duplication of *StSUMO* genes compared within potato and between potato and *Arabidopsis* might be the reason that potato and *Arabidopsis* contain few SUMO genes.

### 3.4. Gene Structure and Motif Analysis

**Figure 4:**
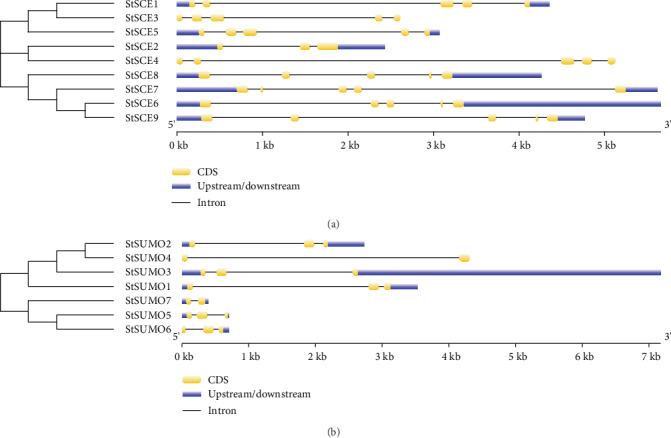
Structure of (a) *StSCE* and (b) *StSUMO* genes potato showing CDS, upstream/downstream, and introns.

Information on the structural diversity of the SCE and SUMO genes in potato was identified by GSDS 2.0 (Figures 4(a) and 4(b)). Numbers of introns ranged from 2 to 4 where eight *StSCE* genes contained 4 and one *StSCE* gene contained 2 introns. The four introns were found in rice *SCE1/2* and *3* genes in the previous study [8], and *AtSUM3* contained 3 introns [40]. In *StSUMO* genes, the number of introns ranged from 1 to 2. *StSUMO4* and *7* contained single intron whereas the remaining 5 StSUMO genes contained two introns. Diversity in the structure of exon and intron is very crucial for the evolution of the multigene family [41]. Progression of the gene family is linked to the diversity of exon and intron structures, and the presence of introns is considered to be responsible for the evolution of the new gene family by alternative splicing and shuffling of exons [42].

**Figure 5:**
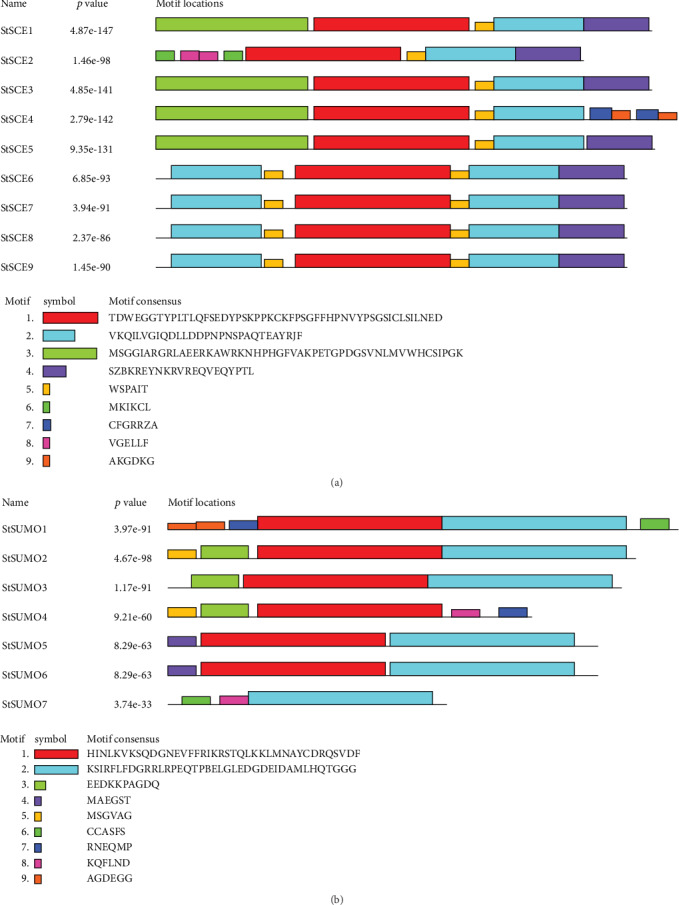
The discovered conserved motifs of (a) *StSCE* and (b) *StSUMO* proteins. Different color boxes indicate different motifs.

The results obtained from MEME were analyzed to discover the conserved motifs in nine SCE and seven StSUMO genes. Nine motifs were discovered in both *StSCE* and *StSUMO* genes (Figures 5(a) and 5(b)). Motifs 1 and 2 in *StSCE* genes were annotated by the Interproscan as RWD/UBC-like domain. All the remaining motifs (3 to 9) of *StSCE* genes and all the motifs of *StSUMO* genes were unknown domains according to Interproscan. The details on conserved motifs discovered from MEME are attached in Supplementary Table 4. The types and numbers of conserved motifs present in the genes varied; however, conserved motifs 1, 2, and 5 were present in all the genes. It means that these three conserved motifs could have similar functions in potato genes. *StSCE2* and *StSCE4* contained six conserved motifs while the other remaining seven *StSCE* genes contained five conserved motifs but motifs 7 and 8 were present only in *StSCE4* and *StSCE2*, respectively. In *StSUMO* genes, the numbers of motifs varied from 3 to 5. Motif 1 was present in all the *StSUMO* genes except *StSUMO7*, while motif 2 was present in all the genes except in *StSUMO4*. The same motifs were present in StSUMO5 and 6, so these two proteins might have similarities in their functions, while the presence of motif numbers and types varied in other genes. *AtSCE* and *AtSUM* genes contained 10 whereas *OsSCE* and *OsSUMO* genes contained 8 different motifs.

### 3.5. Analysis of *cis-Acting* Elements

**Figure 6:**
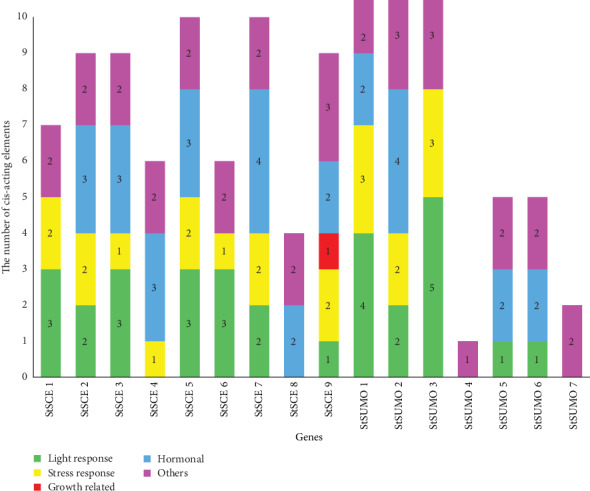
The cis-elements of the promoter region in *StSCE* and *StSUMO* genes of potato were predicted using Plant CARE.

The *cis-acting* elements are describing transcription factors that regulate the expression of genes on the same chromosome [43]. The *cis-acting* elements present in the promoter region of the genes have multiple functions in addition to gene transcription. These elements are also highly involved in the stress response. To further the probable mechanism of *StSCE* and StSUMO genes in the various stress responses, the promoter sequences were submitted to Plant CARE [35] for analysis. Thirty different cis-acting elements with 48 different sequences were identified in *StSCE* genes with known functions. Likewise, StSUMO genes contained 25 different cis-acting elements with 42 different sequences with known functions (Figure 6) (for details, please refer to Supplementary Table 5).

For the in-depth analysis of cis-acting elements, different cis-acting elements were divided into 5 different categories according to their functions. Five categories were light response, growth-related response, stress response, hormonal response, and others. The presence of AE-box (part of a module for light response), Box4 (part of a conserved DNA module involved in light responsiveness), and GATT-motif (part of a light-responsive element) was high in frequency under light response in *StSCE* genes. Similarly, ARE (cis-acting regulatory element essential for the anaerobic induction) in stress-related response, ABRE (cis-acting element involved in the abscisic acid responsiveness) in hormonal response, and CAAT-box (common cis-acting element in promoter and enhancer regions) and TATA-box (core promoter element around -30 of transcription start) in other categories were high in frequency in *StSCE* and *StSUMO* genes.

CAAT-box was present in all the *StSCE* and *StSUMO* genes with common *cis-acting* elements in the promoter and enhancer region function. All the *StSCE* and *StSUMO* genes possessed at least one *cis-acting* element. The highest number of *cis-acting* elements in *StSCE* and *StSUMO* genes was the hormonal response and other category, respectively. The analysis of the promoter regions and *cis-acting* elements revealed that most SCE and SUMO genes contained several replicas of *cis*-elements whose role might be crucial to boosting the regulation for gene transcription ultimately supporting to cope with the adverse environmental conditions.

### 3.6. Analysis of Gene Expression Profile

**Figure 7:**
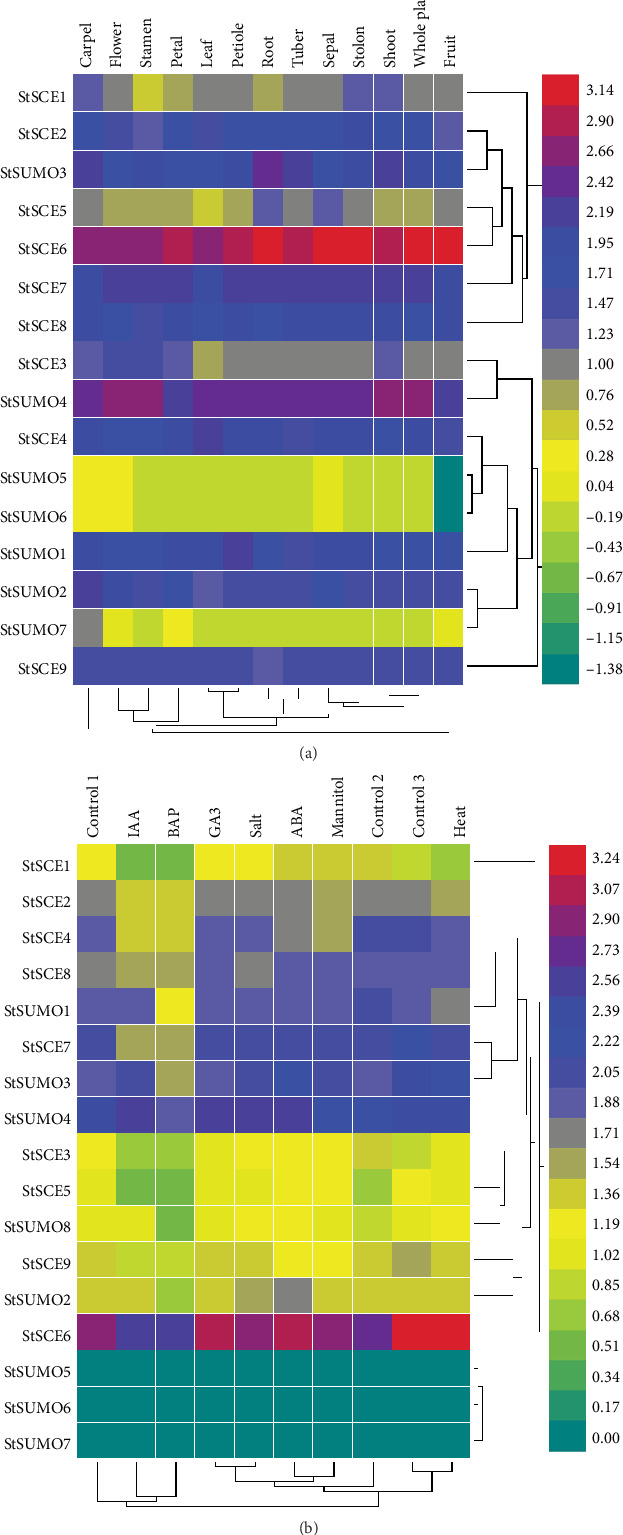
The *StSCE* and *StSUMO* genes expression in different organs (a) and under different treatments (hormonal and abiotic stress) (b). The expression level increases with an increase in color gradient from green to red. FPKM values of the genes were transformed by log10. Control1/2 and 3 are for hormonal, salt and mannitol, heat treatments, respectively.

Heat map of 9 *StSCE* and 7 *StSUMO* genes represented by FPKM values in different tissues and organs using RNA-seq data was visualized by using HemI [39]. The data on gene expression on tissues and organs in normal conditions included carpel, flower, leaf, petal, petiole, root, sepal, shoot, stamen, tuber, stolon, fruit, and whole plant (Figure 7(a)). The expression pattern of the genes is under treatment condition; hormonal (BAP, ABA, IAA, and GA3) and abiotic stress (heat, NaCl, and mannitol) were compared with control (Figure 7(b)).

The analysis showed that all the *StSCE* and *StSUMO* genes were expressed at least in one tissue. For instance, *StSCE6* and *StSUMO4* were predominantly expressed in all tissue and organs especially in roots, sepals, and fruits. *StSCE5*, *StSUMO5*, and *6* were not expressed in most of the tissues and organs.

**Figure 8:**
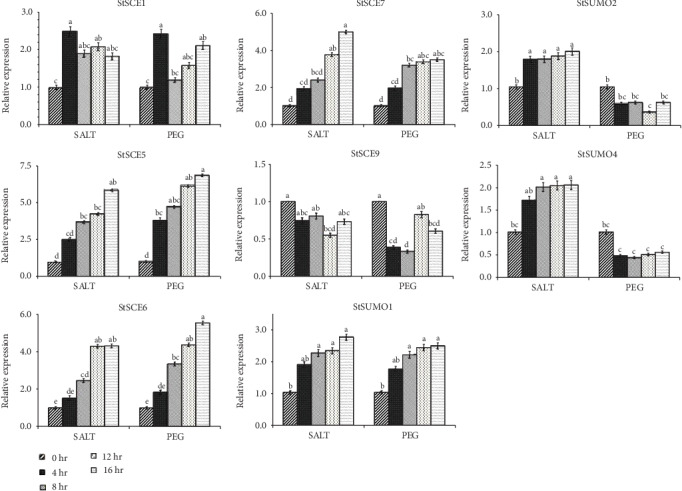
The relative expression of qRT-PCR for *StSCE* and *StSUMO* genes under salt and PEG treatments. Values were log transformed and displayed by means ± standard deviation (SD). Alphabets indicate the relative expression compared to control (0 hr). Bars represent the standard errors from three replicates Duncan's multiple range test (*p* < 0.05).

Most of the genes were overexpressed in ABA treatment. *StSCE5/6/7* and *StSUMO3* were overexpressed whereas *StSCE1/3/4/9* and *StSUMO1* were downregulated in salt- and mannitol-treated conditions. *StSUMO5/6* and *7* did not show any response to any treatments. *StSCE2* and *StSUMO2* and *4* were overexpressed under salt treatment whereas downregulated under mannitol treatment. *StSCE3/6* and *StSUMO4* were overexpressed during heat-treated condition while the other remaining genes were downregulated. The expression pattern study of *StSCE* and StSUMO genes under drought and salt stress conditions was analyzed by qRT-PCR with two technical and three biological replications to obtain the transcript level. *StSCE1/5/6/7* and *9* and *StSUMO1/2* and *4* were selected for qRT-PCR analysis (Figure 8).

The expression was upregulated in *StSCE1/5/6* and *7* under salt stress and PEG treatment except for *StSCE9* which was upregulated in salt and downregulated under PEG treatment. Similarly, *StSUMO1/2* and *4* showed upregulated during salt stress whereas *StSUMO2* and *4* were observed downregulated during PEG treatment.

This study was the preliminary research for exploring *StSCE* and *StSUMO* genes which are the important members of the SUMOylation pathway. During the study, we observed that most of the genes were upregulated during salt and PEG treatment. Study in *Arabidopsis* suggested that SUMOylation is the important mechanism which regulates the normal functioning of plants during abiotic stress condition [44, 45]. Due to the limited findings on the roles of SCE and SUMO genes in potatoes, the further exploration of the mechanism of these genes to regulate during abiotic condition is needed; however, overexpression of *OsSCE1* gene in rice showed hypersensitivity to drought and *OsSCE3* overexpressed rice was tolerance to drought [8]. Among the eight genes taken for qRT-PCR, most of them showed a response towards stress condition, thereby indicating that these genes could play important roles in regulating plant growth during abiotic stress conditions.

## 4. Conclusion

Sixteen genes (*StSCE1-9* and *StSUMO1-7*) in potato were confirmed and comprehensively analyzed. The gene structure, chromosomal localization, gene duplication, and phylogenetic analysis and prediction of *cis-acting* elements were analyzed. *StSCE* and *StSUMO* genes were observed to be induced by the abiotic stress (PEG and salt), so the results suggest that these genes might have crucial roles in the physiological functions. So, the stress-induced genes obtained from the present study can be utilized as appropriate candidates for improving the agronomic traits of potato. This study provides a base for further study which would help to explore the tissue-specific or developmental-specific roles of *StSCE* and *StSUMO* genes.

## Data Availability

The data (Supplementary Files.zip) used to support the findings of this study are included within the supplementary information files.

## Supplementary Materials

Supplementary Materials Table S1: the primer designed for qRT-PCR. Table S2: the StSCE and StSUMO genomics ID, polypeptide, locus, gene name, domain information, chromosomal location, predicted subcellular location(s), PI, Mw, number of amino acids, introns, instability index, aliphatic index, and GRAVY of the StSCE family in potato. Table S3: protein interaction, SUMOylation sites, and SIMS. Table S4: the conserved motifs of StSCE and StSUMO genes in potato. Table S5: the cis-acting elements of StSCE and StSUMO genes.
