# Supplementary material for: Genomic Analysis of the SUMO-Conjugating Enzyme and Genes under Abiotic Stress in Potato (Solanum tuberosum L.)
Source: Int J Genomics. 2020 Jun 24;2020:9703638. doi: 10.1155/2020/9703638 (PMC7335410; doi:10.1155/2020/9703638)
Supplement: Supplementary Materials — Table S1: the primer designed for qRT-PCR. Table S2: the StSCE and StSUMO genomics ID, polypeptide, locus, gene name, domain information, chromosomal location, predicted subcellular location(s), PI, Mw, number of amino acids, introns, instability index, aliphatic index, and GRAVY of the StSCE family in potato. Table S3: protein interaction, SUMOylation sites, and SIMS. Table S4: the conserved motifs of StSCE and StSUMO genes in potato. Table S5: the cis-acting elements of StSCE and StSUMO genes. [file 9703638.f1.zip › 9703638.f1/Supplementary S2 The StSCE and StSUMO genomics ID.docx]

| **Table S2: The *StSCE* and *StSUMO* genomics ID, polypeptide, locus, gene name, Domain information, chromosomal location, predicted subcellular location(s), PI , Mw, number of amino acids, introns, instability index, aliphatic index, GRAVY of StSCE family in potato** | | | | | | | | | | | | | |
| --- | --- | --- | --- | --- | --- | --- | --- | --- | --- | --- | --- | --- | --- |
| **Genomics ID** | **Polypeptide** | **Locus** | **Gene name** | **Domain** | **Amino Acids** | **Intron/Exon** | **Chromosomal location** | **Predicted Subcellular location(s)** | **pI** | **Mw** | **Instability Index** | **Aliphatic Index** | **GRAVY** |
| **PGSC0003DMT400020524** | **PGSC0003DMP400013981** | **PGSC0003DMG400007950** | StSCE1 | UBCc domain | 160aa | 4/7 | Chro4:67867956 -67872308 | Nucleus | 7 | 18013.4 | 51.34 | 61.56 | -0.718 |
| **PGSC0003DMT400025497** | **PGSC0003DMP400017363** | **PGSC0003DMG400009844** | StSCE2 | UBCc domain | 138aa | 2/5 | Chro0: 37640204 -7640204 | Nucleus | 5.9 | 15715.3 | 53.17 | 95.94 | -0.013 |
| **PGSC0003DMT400039840** | **PGSC0003DMP400026999** | **PGSC0003DMG400015405** | StSCE3 | UBCc domain | 160aa | 4/5 | Chro12: 56519864- 56522473 | Nucleus | 8.8 | 17954.4 | 49.98 | 64 | -0.578 |
| **PGSC0003DMT400016581** | **PGSC0003DMP400011490** | **PGSC0003DMG400006481** | StSCE4 | UBCc domain | 168aa | 4/5 | Chro3: 7830165-7835288 | Nucleus | 9 | 18662.2 | 52.34 | 63.33 | -0.562 |
| **PGSC0003DMT400046775** | **PGSC0003DMP400031652** | **PGSC0003DMG400018161** | StSCE5 | UBCc domain | 161aa | 4/7 | Chro3:52589288- 52592358 | Nucleus | 8.9 | 17932.6 | 51.71 | 74.53 | -0.419 |
| **PGSC0003DMT400083067** | **PGSC0003DMP400055790** | **PGSC0003DMG400033039** | StSCE6 | UBCc domain | 152aa | 4/7 | Chro6:51799496-51805149 | Cytoplasm,Nucleus | 5.4 | 17334.5 | 73.04 | 70 | -0.577 |
| **PGSC0003DMT400046571** | **PGSC0003DMP400031504** | **PGSC0003DMG400018082** | StSCE7 | UBCc domain | 152aa | 5/7 | Chro3:53032298- 53037916 | Cytoplasm.Nucleus | 5.4 | 17349.5 | 73.23 | 71.91 | -0.594 |
| **PGSC0003DMT400017858** | **PGSC0003DMP400012280** | **PGSC0003DMG401006935** | StSCE8 | UBCc domain | 151aa | 5/7 | Chro2:25945963-25950226 | Cytoplasm.Nucleus | 5.9 | 17186.4 | 66.38 | 71.32 | -0.539 |
| **PGSC0003DMT400046771** | **PGSC0003DMP400031650** | **PGSC0003DMG402018159** | StSCE9 | UBCc domain | 152aa | 4/6 | Chro3:52628876- 52633639 | Cytoplasm.Nucleus | 5.4 | 17343.5 | 68.3 | 68.68 | -0.578 |
| **PGSC0003DMT400059463** | **PGSC0003DMP400040020** | **PGSC0003DMG400023103** | StSUMO1 | Rad60-SLD | 108aa | 2/5 | chro9: 43,036,513- 43,040,040 | Nucleus | 4.81 | 12072.43 | 37.64 | 53.33 | -0.738 |
| **PGSC0003DMT400057163** | **PGSC0003DMP400038444** | **PGSC0003DMG400022207** | StSUMO2 | Rad60-SLD | 99aa | 2/5 | Chro7: 55,064,671 -55,067,397 | Nucleus | 4.95 | 11178.44 | 54.36 | 65.05 | -0.825 |
| **PGSC0003DMT400001775** | **PGSC0003DMP400001311** | **PGSC0003DMG400000665** | StSUMO3 | Rad60-SLD | 96aa | 2/5 | Chro7: 45,136,637 - 45,139,322 | Cytoplasm ,Nucleus | 4.93 | 10908.15 | 51.63 | 65.00 | -0.844 |
| **PGSC0003DMT400036088** | **PGSC0003DMP400024516** | **PGSC0003DMG400013899** | StSUMO4 | Rad60-SLD | 77aa | 1/2 | chro12: 6,384,758 - 6,389,067 | Nulceus | 8.89 | 8648.71 | 61.61 | 62.08 | -0.810 |
| **PGSC0003DMT400080380** | **PGSC0003DMP400054531** | **PGSC0003DMG400031298** | StSUMO5 | Rad60-SLD | 91aa | 2/4 | chro9: 58,878,346- 58,879,048 | Nulceus | 9.41 | 10390.09 | 34.92 | 66.48 | -0.476 |
| **PGSC0003DMT400078111** | **PGSC0003DMP400052882** | **PGSC0003DMG400030378** | StSUMO6 | Rad60-SLD | 91aa | 2/4 | chro9: 58,878,346- 58,879,048 | Nulceus | 9.41 | 10390.09 | 34.92 | 66.48 | -0.476 |
| **PGSC0003DMT400080379** | **PGSC0003DMP400054530** | **PGSC0003DMG400031297** | StSUMO7 | Rad60-SLD | 59aa | 1/4 | Chro9:58,876,160 to 58,876,550 | Mitochondria | 6.54 | 6797.66 | 54.24 | 71.02 | -0.671 |
