# Supplementary material for: Genomic Analysis of the SUMO-Conjugating Enzyme and Genes under Abiotic Stress in Potato (Solanum tuberosum L.)
Source: Int J Genomics. 2020 Jun 24;2020:9703638. doi: 10.1155/2020/9703638 (PMC7335410; doi:10.1155/2020/9703638)
Supplement: Supplementary Materials — Table S1: the primer designed for qRT-PCR. Table S2: the StSCE and StSUMO genomics ID, polypeptide, locus, gene name, domain information, chromosomal location, predicted subcellular location(s), PI, Mw, number of amino acids, introns, instability index, aliphatic index, and GRAVY of the StSCE family in potato. Table S3: protein interaction, SUMOylation sites, and SIMS. Table S4: the conserved motifs of StSCE and StSUMO genes in potato. Table S5: the cis-acting elements of StSCE and StSUMO genes. [file 9703638.f1.zip › 9703638.f1/Supplementary S3 SUMOylation sites and SIMs.docx]

**Table S3: Protein interaction, SUMOylation Sites and SIMS**

| **ID** | **Position** | **Peptide** | **P-Value** | **Type** | **Protein interaction** |
| --- | --- | --- | --- | --- | --- |
| StSCE1 | 15 | GRLAEERKAWRKNHP | 0.162 | Sumoylation Nonconcensus | PGSC0003DMT400078207, PGSC0003DMT400079486,  StSUMO1,  StSUMO2,  StSUMO3 |
|  | 19 | EERKAWRKNHPHGFV | 0.939 | Sumoylation Nonconcensus |  |
|  | 28 | HPHGFVAKPETGPDG | 0.03 | Sumoylation Concensus |  |
|  | 37 - 41 | ETGPDGT VNLMV WQCSIPG | 0.829 | SUMO Interaction |  |
|  | 49 | WQCSIPGKPSTDWEG | 0.977 | Sumoylation Nonconcensus |  |
|  | 72 | FSEDYPSKPPKCKFP | 0.924 | Sumoylation Nonconcensus |  |
|  | 75 | DYPSKPPKCKFPAGF | 0.789 | Sumoylation Nonconcensus |  |
|  | 77 | PSKPPKCKFPAGFFH | 0.908 | Sumoylation Nonconcensus |  |
|  | 95 - 99 | YPSGTVC LSILN EDSGWRP | 0.69 | SUMO Interaction |  |
|  | 111 | WRPAITVKQILVGIQ | 0.825 | Sumoylation Nonconcensus |  |
|  | 113 - 117 | PAITVKQ ILVGI QDLLDQP | 0.212 | SUMO Interaction |  |
|  | 146 | MQDTDEYKKRVKQQA | 0.051 | Sumoylation Nonconcensus |  |
|  | 147 | QDTDEYKKRVKQQAK | 0.153 | Sumoylation Nonconcensus |  |
|  | 150 | DEYKKRVKQQAKQYP | 0.054 | Sumoylation Nonconcensus |  |
|  | 154 | KRVKQQAKQYPALL* | 0.047 | Sumoylation Nonconcensus |  |
| StSCE2 | 2 | ******MKVLCLSDV | 0.892 | Sumoylation Nonconcensus |  |
|  | 3 to 7 | *****MK VLCLS DVGELVF | 0.819 | SUMO Interaction |  |
|  | 12 to 16 | CLSDVGE LVFVF LLLFLLT | 0.624 | SUMO Interaction |  |
|  | 18 - 22 | ELVFVFL LLFLL TTIKCLQ | 0.865 | SUMO Interaction |  |
|  | 26 | LFLLTTIKCLQTDWE | 0.926 | Sumoylation Nonconcensus |  |
|  | 50 | FSEDYPSKPPKCKFP | 0.991 | Sumoylation Nonconcensus |  |
|  | 53 | DYPSKPPKCKFPPGF | 0.844 | Sumoylation Nonconcensus |  |
|  | 55 | PSKPPKCKFPPGFFH | 0.952 | Sumoylation Nonconcensus |  |
|  | 73 - 77 | YPSGTVC LSILN EDSGWRP | 0.69 | SUMO Interaction |  |
|  | 89 | WRPAITVKQILVGIQ | 0.886 | Sumoylation Nonconcensus |  |
|  | 91 - 95 | PAITVKQ ILVGI QDLLDQP | 0.212 | SUMO Interaction |  |
|  | 117 - 121 | TEGYHMF IQDVL EYRKRVR | 0.479 | SUMO Interaction |  |
|  | 125 | QDVLEYRKRVRLQSK | 0.363 | Sumoylation Nonconcensus |  |
|  | 132 | KRVRLQSKQYPPLV* | 0.049 | Sumoylation Nonconcensus |  |
| StSCE3 | 15 | GRLAEERKSWRKNHP | 0.085 | Sumoylation Nonconcensus | PGSC0003DMT400078207,  PGSC0003DMT400079486,  StSUMO1,  StSUMO2,  StSUMO3 |
|  | 19 | EERKSWRKNHPHGFV | 0.966 | Sumoylation Nonconcensus |  |
|  | 37 - 41 | ETGADGS VNLMV WNCSIPG | 0.834 | SUMO Interaction |  |
|  | 49 | WNCSIPGKAKTDWEG | 0.953 | Sumoylation Nonconcensus |  |
|  | 51 | CSIPGKAKTDWEGGF | 0.073 | Sumoylation Concensus |  |
|  | 72 | FSEDFPSKPPKCKFP | 0.966 | Sumoylation Nonconcensus |  |
|  | 75 | DFPSKPPKCKFPQGF | 0.745 | Sumoylation Nonconcensus |  |
|  | 77 | PSKPPKCKFPQGFFH | 0.913 | Sumoylation Nonconcensus |  |
|  | 95 - 99 | YPSGTIC LSILN EDSGWRP | 0.713 | SUMO Interaction |  |
|  | 111 | WRPAITVKQILVGIQ | 0.793 | Sumoylation Nonconcensus |  |
|  | 113 - 117 | PAITVKQ ILVGI QDLLVQP | 0.229 | SUMO Interaction |  |
|  | 120 - 124 | ILVGIQD LLVQP NPSDPAQ | 0.727 | SUMO Interaction |  |
|  | 146 | MQDGAEYKKRVRQQA | 0.05 | Sumoylation Nonconcensus |  |
|  | 147 | QDGAEYKKRVRQQAK | 0.137 | Sumoylation Nonconcensus |  |
|  | 154 | KRVRQQAKQYPALV* | 0.046 | Sumoylation Nonconcensus |  |
| StSCE4 | 15 | GRLAEERKAWRKNHP | 0.177 | Sumoylation Nonconcensus | PGSC0003DMT400078207, PGSC0003DMT400079486,  StSUMO1,  StSUMO2,  StSUMO3 |
|  | 19 | EERKAWRKNHPHGFV | 0.967 | Sumoylation Nonconcensus |  |
|  | 28 | HPHGFVAKPETLPDG | 0.026 | Sumoylation Concensus |  |
|  | 37 - 41 | ETLPDGS VNLMV WHCSIPG | 0.834 | SUMO Interaction |  |
|  | 49 | WHCSIPGKTGTDWEG | 0.965 | Sumoylation Nonconcensus |  |
|  | 72 | FSEDYPSKPPKCKFP | 0.971 | Sumoylation Nonconcensus |  |
|  | 75 | DYPSKPPKCKFPQGF | 0.82 | Sumoylation Nonconcensus |  |
|  | 77 | PSKPPKCKFPQGFFH | 0.947 | Sumoylation Nonconcensus |  |
|  | 95 - 99 | YPSGTVC LSILN EDSGWRP | 0.69 | SUMO Interaction |  |
|  | 111 | WRPAITVKQILVGIQ | 0.886 | Sumoylation Nonconcensus |  |
|  | 113 - 117 | PAITVKQ ILVGI QDLLDQP | 0.212 | SUMO Interaction |  |
|  | 149 | FGRREATKGDKGLPR | 0.132 | Sumoylation Nonconcensus |  |
|  | 152 | REATKGDKGLPRHAA | 0.291 | Sumoylation Nonconcensus |  |
| StSCE5 | 15 | GRLAEERKAWRKNHP | 0.188 | Sumoylation Nonconcensus | PGSC0003DMT400078207, PGSC0003DMT400079486,  StSUMO1,  StSUMO2,  StSUMO3 |
|  | 19 | EERKAWRKNHPHGFV | 0.861 | Sumoylation Nonconcensus |  |
|  | 49 | WRCIIPGKPGTDWEG | 0.969 | Sumoylation Nonconcensus |  |
|  | 61 - 65 | WEGGHYP LIMLF SEDYPSQ | 0.422 | SUMO Interaction |  |
|  | 75 | DYPSQPPKCKFPPGF | 0.79 | Sumoylation Nonconcensus |  |
|  | 77 | PSQPPKCKFPPGFFH | 0.88 | Sumoylation Nonconcensus |  |
|  | 95 - 99 | YPSGDVC LSILN TGLGWSP | 0.649 | SUMO Interaction |  |
|  | 114 - 118 | AITVTQI LVGIQ ELLDEPN | 0.167 | SUMO Interaction |  |
|  | 136 | SAQFECHKLYVQKDK | 0.559 | Sumoylation Nonconcensus |  |
|  | 141 | CHKLYVQKDKTEYKK | 0.096 | Sumoylation Nonconcensus |  |
|  | 143 | KLYVQKDKTEYKKRV | 0.05 | Sumoylation Nonconcensus |  |
|  | 147 | QKDKTEYKKRVKAQA | 0.048 | Sumoylation Nonconcensus |  |
|  | 148 | KDKTEYKKRVKAQAK | 0.133 | Sumoylation Nonconcensus |  |
|  | 151 | TEYKKRVKAQAKQYP | 0.052 | Sumoylation Nonconcensus |  |
|  | 155 | KRVKAQAKQYPALL* | 0.046 | Sumoylation Nonconcensus |  |
| StSCE6 | 7 | *MSTPARKRLMRDFK | 0.666 | Sumoylation Nonconcensus | PGSC0003DMT400078207,  PGSC0003DMT400079486,  StSUMO1,  StSUMO2,  StSUMO3 |
|  | 14 | KRLMRDFKRLQQDPP | 0.122 | Sumoylation Nonconcensus |  |
|  | 54 | PWDGGTFKLTLQFSE | 0.835 | Sumoylation Nonconcensus |  |
|  | 66 | FSEDYPNKPPTVRFV | 0.939 | Sumoylation Nonconcensus |  |
|  | 89 - 93 | YADGSIC LDILQ NQWSPIY | 0.596 | SUMO Interaction |  |
|  | 102 - 106 | QWSPIYD VAAIL TSIQSLL | 0.468 | SUMO Interaction |  |
|  | 109 - 113 | VAAILTS IQSLL CDPNPNS | 0.364 | SUMO Interaction |  |
|  | 134 | ARMFSENKREYNRRV | 0.045 | Sumoylation Nonconcensus |  |
|  | 141 - 145 | KREYNRR VREIV EQSWTAD | 0.074 | SUMO Interaction |  |
| StSCE7 | 7 | *MSTPSRKRLMRDFK | 0.733 | Sumoylation Nonconcensus | PGSC0003DMT400078207,  PGSC0003DMT400079486,  StSUMO1,  StSUMO2,  StSUMO3 |
|  | 14 | KRLMRDFKRLQQDPP | 0.122 | Sumoylation Nonconcensus |  |
|  | 54 | SWDGGTFKLTLQFNE | 0.816 | Sumoylation Nonconcensus |  |
|  | 66 | FNEDYPNKPPTVRFV | 0.939 | Sumoylation Nonconcensus |  |
|  | 89 - 93 | YADGSIC LDILQ NQWSPIY | 0.596 | SUMO Interaction |  |
|  | 102 - 106 | QWSPIYD VAAIL TSIQSLL | 0.468 | SUMO Interaction |  |
|  | 109 - 113 | VAAILTS IQSLL CDPNPNS | 0.447 | SUMO Interaction |  |
|  | 134 | ARLFSENKREYNRRV | 0.045 | Sumoylation Nonconcensus |  |
|  | 141 - 145 | KREYNRR VREIV EQSWTAD | 0.074 | SUMO Interaction |  |
| StSCE8 | 6 | **MSTAAKKRLMRDF | 0.19 | Sumoylation Nonconcensus | PGSC0003DMT400078207, PGSC0003DMT400079486,  StSUMO1,  StSUMO2,  StSUMO3 |
|  | 7 | *MSTAAKKRLMRDFK | 0.583 | Sumoylation Nonconcensus |  |
|  | 14 | KRLMRDFKRLQQDPP | 0.15 | Sumoylation Nonconcensus |  |
|  | 54 | AWDGGTFKMTLQFSE | 0.852 | Sumoylation Nonconcensus |  |
|  | 66 | FSEDYPNKPPKVHFV | 0.967 | Sumoylation Nonconcensus |  |
|  | 69 | DYPNKPPKVHFVSRM | 0.991 | Sumoylation Nonconcensus |  |
|  | 89 - 93 | YADGSIC LDILQ NQWSPIY | 0.596 | SUMO Interaction |  |
|  | 102 - 106 | QWSPIYD VAAIL TSIQSLL | 0.473 | SUMO Interaction |  |
|  | 109 - 113 | VAAILTS IQSLL SDPNPNS | 0.458 | SUMO Interaction |  |
|  | 134 | ARLFSENKRDYNRKV | 0.05 | Sumoylation Nonconcensus |  |
|  | 140 | NKRDYNRKVREIVEQ | 0.053 | Sumoylation Nonconcensus |  |
|  | 141 - 145 | KRDYNRK VREIV EQSWTAD | 0.082 | SUMO Interaction |  |
| StSCE9 | 7 | *MSTPSRKRLMRDFK | 0.722 | Sumoylation Nonconcensus | PGSC0003DMT400078207, PGSC0003DMT400079486,  StSUMO1,  StSUMO2,  StSUMO3 |
|  | 14 | KRLMRDFKRLQQDPP | 0.115 | Sumoylation Nonconcensus |  |
|  | 54 | PWDGGTFKLTLQFSE | 0.835 | Sumoylation Nonconcensus |  |
|  | 66 | FSEDYPNKPPTVRFV | 0.939 | Sumoylation Nonconcensus |  |
|  | 89 - 93 | YADGSIC LDILQ NQWSPIY | 0.596 | SUMO Interaction |  |
|  | 102 - 106 | QWSPIYD VAAIL TSIQSLL | 0.468 | SUMO Interaction |  |
|  | 109 - 113 | VAAILTS IQSLL CDPNPNS | 0.364 | SUMO Interaction |  |
|  | 134 | ARMFSENKREYNRKV | 0.046 | Sumoylation Nonconcensus |  |
|  | 140 | NKREYNRKVREVVEQ | 0.053 | Sumoylation Nonconcensus |  |
|  | 141 - 145 | KREYNRK VREVV EQSWTAD | 0.102 | SUMO Interaction |  |
| StSUMO1 | 12 | GGAGDEDKKPNDQMV | 0.886 | Sumoylation Nonconcensus | PGSC0003DMT400020962, PGSC0003DMT400025497, PGSC0003DMT400046775, PGSC0003DMT400079486,  StSCE3,  StSUMO2,  StSUMO3 |
|  | 13 | GAGDEDKKPNDQMVH | 0.351 | Sumoylation Nonconcensus |  |
|  | 21 - 25 | PNDQMVH INLKV KGQDGNE | 0.951 | SUMO Interaction |  |
|  | 24 | QMVHINLKVKGQDGN | 0.12 | Sumoylation Nonconcensus |  |
|  | 26 | VHINLKVKGQDGNEV | 0.92 | Sumoylation Nonconcensus |  |
|  | 38 | NEVFFRIKRSTQMRK | 0.259 | Sumoylation Nonconcensus |  |
|  | 45 | KRSTQMRKLMNAYCD | 0.111 | Sumoylation Nonconcensus |  |
| StSUMO2 | 11 | VTQQEEEKKPAADQG | 0.09 | Sumoylation Nonconcensus | PGSC0003DMT400020962, PGSC0003DMT400025497, PGSC0003DMT400046775, PGSC0003DMT400079486,  StSCE3,  StSUMO1,  StSUMO3 |
|  | 12 | TQQEEEKKPAADQGG | 0.189 | Sumoylation Nonconcensus |  |
|  | 21 - 25 | AADQGGH INLKV KSQDGNE | 0.937 | SUMO Interaction |  |
|  | 24 | QGGHINLKVKSQDGN | 0.128 | Sumoylation Nonconcensus |  |
|  | 26 | GHINLKVKSQDGNEV | 0.896 | Sumoylation Nonconcensus |  |
|  | 38 | NEVFFRIKRSTQLKK | 0.342 | Sumoylation Nonconcensus |  |
|  | 44 | IKRSTQLKKLMNAYC | 0.343 | Sumoylation Nonconcensus |  |
|  | 45 | KRSTQLKKLMNAYCD | 0.245 | Sumoylation Nonconcensus |  |
| StSUMO3 | 9 | SQTAEEDKKPGGDQI | 0.75 | Sumoylation Nonconcensus | PGSC0003DMT400020962, PGSC0003DMT400025497, PGSC0003DMT400046775, PGSC0003DMT400079486,  StSCE3,  StSUMO1,  StSUMO2 |
|  | 10 | QTAEEDKKPGGDQIH | 0.269 | Sumoylation Nonconcensus |  |
|  | 16 - 20 | KKPGGDQ IHINL KVKSQDG | 0.923 | SUMO Interaction |  |
|  | 21 | DQIHINLKVKSQDGN | 0.128 | Sumoylation Nonconcensus |  |
|  | 23 | IHINLKVKSQDGNEV | 0.896 | Sumoylation Nonconcensus |  |
|  | 35 | NEVFFRIKRSTQLKK | 0.342 | Sumoylation Nonconcensus |  |
|  | 41 | IKRSTQLKKLMNAYC | 0.343 | Sumoylation Nonconcensus |  |
|  | 42 | KRSTQLKKLMNAYCD | 0.245 | Sumoylation Nonconcensus |  |
| StSUMO4 | 11 | VAGGEEDKKPAGDQS | 0.168 | Sumoylation Nonconcensus | PGSC0003DMT400020962, PGSC0003DMT400025497, PGSC0003DMT400046775, PGSC0003DMT400079486,  StSCE3 |
|  | 12 | AGGEEDKKPAGDQSG | 0.45 | Sumoylation Nonconcensus |  |
|  | 21 - 25 | AGDQSGH INLKV KSQDGNE | 0.935 | SUMO Interaction |  |
|  | 24 | QSGHINLKVKSQDGN | 0.128 | Sumoylation Nonconcensus |  |
|  | 26 | GHINLKVKSQDGNEV | 0.896 | Sumoylation Nonconcensus |  |
|  | 38 | NEVFFRIKRSTQLKK | 0.342 | Sumoylation Nonconcensus |  |
|  | 44 | IKRSTQLKKLMNAYC | 0.343 | Sumoylation Nonconcensus |  |
|  | 45 | KRSTQLKKLMNAYCD | 0.245 | Sumoylation Nonconcensus |  |
| StSUMO5 | 7 | *MAEGSTKFIKLKIK | 0.532 | Sumoylation Nonconcensus | PGSC0003DMT400020962, PGSC0003DMT400025497, PGSC0003DMT400046775, PGSC0003DMT400079486,  StSCE3 |
|  | 9 to 13 | AEGSTKF IKLKI KAQDDTI | 0.986 | SUMO Interaction |  |
|  | 10 | EGSTKFIKLKIKAQD | 0.135 | Sumoylation Nonconcensus |  |
|  | 12 | STKFIKLKIKAQDDT | 0.178 | Sumoylation Nonconcensus |  |
|  | 14 | KFIKLKIKAQDDTIL | 0.922 | Sumoylation Nonconcensus |  |
|  | 24 | DDTILHFKVNPSTIM | 0.7 | Sumoylation Nonconcensus |  |
|  | 32 | VNPSTIMKDIFMSYS | 0.461 | Sumoylation Nonconcensus |  |
|  | 41 | IFMSYSSKKQMMNYK | 0.69 | Sumoylation Nonconcensus |  |
|  | 42 | FMSYSSKKQMMNYKI | 0.259 | Sumoylation Nonconcensus |  |
|  | 48 | KKQMMNYKIFRFFFD | 0.531 | Sumoylation Nonconcensus |  |
|  | 57 | FRFFFDGKRLSPKKT | 0.82 | Sumoylation Nonconcensus |  |
|  | 62 | DGKRLSPKKTVNELG | 0.754 | Sumoylation Nonconcensus |  |
|  | 63 | GKRLSPKKTVNELGL | 0.884 | Sumoylation Nonconcensus |  |
|  | 71 | TVNELGLKNGDEIDA | 0.139 | Sumoylation Nonconcensus |  |
| StSUMO6 | 7 | *MAEGSTKFIKLKIK | 0.532 | Sumoylation Nonconcensus | PGSC0003DMT400020962, PGSC0003DMT400025497, PGSC0003DMT400046775, PGSC0003DMT400079486,  StSCE3 |
|  | 9 to 13 | AEGSTKF IKLKI KAQDDTI | 0.986 | SUMO Interaction |  |
|  | 10 | EGSTKFIKLKIKAQD | 0.135 | Sumoylation Nonconcensus |  |
|  | 12 | STKFIKLKIKAQDDT | 0.178 | Sumoylation Nonconcensus |  |
|  | 14 | KFIKLKIKAQDDTIL | 0.922 | Sumoylation Nonconcensus |  |
|  | 24 | DDTILHFKVNPSTIM | 0.7 | Sumoylation Nonconcensus |  |
|  | 32 | VNPSTIMKDIFMSYS | 0.461 | Sumoylation Nonconcensus |  |
|  | 41 | IFMSYSSKKQMMNYK | 0.69 | Sumoylation Nonconcensus |  |
|  | 42 | FMSYSSKKQMMNYKI | 0.259 | Sumoylation Nonconcensus |  |
|  | 48 | KKQMMNYKIFRFFFD | 0.531 | Sumoylation Nonconcensus |  |
|  | 57 | FRFFFDGKRLSPKKT | 0.82 | Sumoylation Nonconcensus |  |
|  | 62 | DGKRLSPKKTVNELG | 0.754 | Sumoylation Nonconcensus |  |
|  | 63 | GKRLSPKKTVNELGL | 0.884 | Sumoylation Nonconcensus |  |
|  | 71 | TVNELGLKNGDEIDA | 0.139 | Sumoylation Nonconcensus |  |
| StSUMO7 | 2 | ******MKRIFMSYS | 0.429 | Sumoylation Nonconcensus |  |
|  | 12 | FMSYSERKQILNYKT | 0.417 | Sumoylation Nonconcensus |  |
|  | 18 | RKQILNYKTVRFLYN | 0.849 | Sumoylation Nonconcensus |  |
