# Supplementary material for: Genomic Analysis of the SUMO-Conjugating Enzyme and Genes under Abiotic Stress in Potato (Solanum tuberosum L.)
Source: Int J Genomics. 2020 Jun 24;2020:9703638. doi: 10.1155/2020/9703638 (PMC7335410; doi:10.1155/2020/9703638)
Supplement: Supplementary Materials — Table S1: the primer designed for qRT-PCR. Table S2: the StSCE and StSUMO genomics ID, polypeptide, locus, gene name, domain information, chromosomal location, predicted subcellular location(s), PI, Mw, number of amino acids, introns, instability index, aliphatic index, and GRAVY of the StSCE family in potato. Table S3: protein interaction, SUMOylation sites, and SIMS. Table S4: the conserved motifs of StSCE and StSUMO genes in potato. Table S5: the cis-acting elements of StSCE and StSUMO genes. [file 9703638.f1.zip › 9703638.f1/Supplementary S5 cis acting elements.docx]

**Table S5: The cis acting elements of StSCE and StSUMO genes**

| **Response** |  | **cis-elements** | **Sequence** | **Functions** | **StSCE1** | **StSCE2** | **StSCE3** | **StSCE4** | **StSCE5** | **StSCE6** | **StSCE7** | **StSCE8** | **StSCE9** |
| --- | --- | --- | --- | --- | --- | --- | --- | --- | --- | --- | --- | --- | --- |
| Light Response | 17 | AE-box | AGAAACAA | part of a module for light response | 1 | 1 | 1 |  |  | 1 | 1 |  |  |
|  |  | Sp1 | GGGCGG | light responsive element | 1 |  |  |  |  |  |  |  |  |
|  |  | G-box | TAACACGTAG | cis-acting regulatory element involved in light responsiveness |  | 1 |  |  |  |  |  |  |  |
|  |  |  | TACGTG | cis-acting regulatory element involved in light responsiveness |  |  |  |  |  |  |  |  |  |
|  |  | G-Box | CACGTG | cis-acting regulatory element involved in light responsiveness |  |  | 1 |  | 1 |  |  |  |  |
|  |  | G-box | CACGTG | cis-acting regulatory element involved in light responsiveness |  |  | 1 |  |  |  |  |  |  |
|  |  | Box 4 | ATTAAT | part of a conserved DNA module involved in light responsiveness |  |  |  |  | 1 |  |  |  | 1 |
|  |  | TCT-motif | TCTTAC | part of a light responsive element | 1 |  |  |  | 1 |  |  |  |  |
|  |  | GATA-motif | AAGGATAAGG | part of a light responsive element |  |  |  |  |  | 1 |  |  |  |
|  |  | MRE | AACCTAA | MYB binding site involved in light responsiveness |  |  |  |  |  | 1 |  |  |  |
|  |  | Gap-box | CAAATGAA(A/G)A | part of a light responsive element |  |  |  |  |  |  | 1 |  |  |
| Growth related | 1 | MSA-like | TCCAACGGT | cis-acting element involved in cell cycle regulation |  |  |  |  |  |  |  |  | 1 |
| Stress | 19 | ARE | AAACCA | cis-acting regulatory element essential for the anaerobic induction | 1 | 1 | 1 | 1 | 1 | 1 | 1 |  |  |
|  |  | GC-motif | CCCCCG | enhancer-like element involved in anoxic specific inducibility | 1 |  |  |  | 1 |  |  |  |  |
|  |  | LTR | CCGAAA | cis-acting element involved in low-temperature responsiveness |  | 1 |  |  |  |  |  |  | 1 |
|  |  | TC-rich repeats | ATTCTCTAAC | cis-acting element involved in defense and stress responsiveness |  |  |  |  |  |  | 1 |  | 1 |
| Hormone response | 19 | ABRE | ACGTG | cis-acting element involved in the abscisic acid responsiveness |  | 1 | 1 | 1 | 1 |  |  |  |  |
|  |  | P-box | CCTTTTG | gibberellin-responsive element |  | 1 |  | 1 |  |  |  |  |  |
|  |  | TGA-element | AACGAC | auxin-responsive element |  | 1 |  |  |  |  |  |  |  |
|  |  | O2-site | GATGATGTGG | cis-acting regulatory element involved in zein metabolism regulation |  |  | 1 | 1 | 1 |  |  |  |  |
|  |  | TGACG-motif | TGACG | cis-acting regulatory element involved in the MeJA-responsiveness |  |  | 1 |  |  |  | 1 | 1 |  |
|  |  | TCA-element | CCATCTTTTT | cis-acting element involved in salicylic acid responsiveness |  |  |  |  | 1 |  | 1 |  |  |
|  |  | CGTCA-motif | CGTCA | cis-acting regulatory element involved in the MeJA-responsiveness |  |  |  |  |  |  | 1 | 1 |  |
|  |  | TATC-box | TATCCCA | cis-acting element involved in gibberellin-responsiveness |  |  |  |  |  |  | 1 |  | 1 |
|  |  | GARE-motif | TCTGTTG | gibberellin-responsive element |  |  |  |  |  |  |  |  | 1 |
| Others | 19 | CAAT-box | CAAAT | common cis-acting element in promoter and enhancer regions | 1 | 1 | 1 | 1 | 1 | 1 | 1 | 1 | 1 |
|  |  |  | CCAAT | common cis-acting element in promoter and enhancer regions |  |  |  |  |  |  |  |  |  |
|  |  |  | CAAT | common cis-acting element in promoter and enhancer regions |  |  |  |  |  |  |  |  |  |
|  |  |  | TGCCAAC | common cis-acting element in promoter and enhancer regions |  |  |  |  |  |  |  |  |  |
|  |  | TATA-box | ccTATAAAaa | core promoter element around -30 of transcription start | 1 | 1 | 1 |  | 1 | 1 | 1 | 1 |  |
|  |  |  | TATA | core promoter element around -30 of transcription start |  |  |  |  |  |  |  |  |  |
|  |  |  | TATAAA | core promoter element around -30 of transcription start |  |  |  |  |  |  |  |  |  |
|  |  |  | TATACA | core promoter element around -30 of transcription start |  |  |  |  |  |  |  |  |  |
|  |  |  | TACAAAA | core promoter element around -30 of transcription start |  |  |  |  |  |  |  |  |  |
|  |  |  | TATAAAA | core promoter element around -30 of transcription start |  |  |  |  |  |  |  |  |  |
|  |  |  | TATAA | core promoter element around -30 of transcription start |  |  |  |  |  |  |  |  |  |
|  |  |  | TCTTAC | core promoter element around -30 of transcription start |  |  |  |  |  |  |  |  | 1 |
|  |  |  | TACATAAA | core promoter element around -30 of transcription start |  |  |  |  |  |  |  |  |  |
|  |  |  | TATAAATA | core promoter element around -30 of transcription start |  |  |  |  |  |  |  |  |  |
|  |  |  | ATATAA | core promoter element around -30 of transcription start |  |  |  |  |  |  |  |  |  |
|  |  |  | ATTATA | core promoter element around -30 of transcription start |  |  |  |  |  |  |  |  |  |
|  |  |  | TATAAAT | core promoter element around -30 of transcription start |  |  |  |  |  |  |  |  |  |
|  |  |  | ATATAT | core promoter element around -30 of transcription start |  |  |  |  |  |  |  |  |  |
|  |  |  | taTATAAAtc | core promoter element around -30 of transcription start |  |  |  |  |  |  |  |  |  |
|  |  | A-box | CCGTCC | cis-acting regulatory element |  |  |  | 1 |  |  |  |  |  |
|  |  | CCAAT-box | CAACGG | MYBHv1 binding site |  |  |  |  |  |  |  |  |  |
|  |  | circadian | CAAAGATATC | cis-acting regulatory element involved in circadian control |  |  |  |  |  |  |  |  | 1 |
|  |  |  |  |  |  |  |  |  |  |  |  |  |  |
|  |  |  |  |  |  |  |  |  |  |  |  |  |  |
| **Response** |  | **cis-elements** | **Sequence** | **Functions** | **StSUMO1** | **StSUMO2** | **StSUMO3** | **StSUMO4** | **StSUMO5** | **StSUMO6** | **StSUMO7** |  |  |
| Light | 16 | Sp1 | GGGCGG | light responsive element | 1 |  |  |  |  |  |  |  |  |
|  |  | G-box | CACGTG | cis-acting regulatory element involved in light responsiveness |  |  | 1 |  |  |  |  |  |  |
|  |  | Box 4 | ATTAAT | part of a conserved DNA module involved in light responsiveness |  |  | 1 |  | 1 | 1 |  |  |  |
|  |  | GATA-motif | AAGGATAAGG | part of a light responsive element | 1 |  | 1 |  |  |  |  |  |  |
|  |  | GATT-motif | CTCCTGATTAGC | part of a light responsive element | 1 | 1 | 1 |  |  |  |  |  |  |
|  |  | GT1-motif | GGTTAA | light responsive element | 1 | 1 |  |  |  |  |  |  |  |
|  |  | GA-motif | ATAGATAA | part of a light responsive element |  |  | 1 |  |  |  |  |  |  |
|  |  | LAMP-element | CTTTATCA | part of a light responsive element |  |  |  |  |  |  |  |  |  |
| Stress | 11 | ARE | AAACCA | cis-acting regulatory element essential for the anaerobic induction | 1 | 1 | 1 |  |  |  |  |  |  |
|  |  | LTR | CCGAAA | cis-acting element involved in low-temperature responsiveness | 1 |  |  |  |  |  |  |  |  |
|  |  | MBS | CAACTG | MYB binding site involved in drought-inducibility | 1 | 1 | 1 |  |  |  |  |  |  |
|  |  | ATC-motif | AGTAATCT | part of a conserved DNA module involved in light responsiveness |  |  | 1 |  |  |  |  |  |  |
| Hormone response | 15 | ABRE | ACGTG | cis-acting element involved in the abscisic acid responsiveness |  |  |  |  | 1 | 1 |  |  |  |
|  |  | P-box | CCTTTTG | gibberellin-responsive element |  | 1 |  |  |  |  |  |  |  |
|  |  | TGA-element | AACGAC | auxin-responsive element |  |  |  |  |  |  |  |  |  |
|  |  | O2-site | GATGATGTGG | cis-acting regulatory element involved in zein metabolism regulation |  |  |  |  |  |  |  |  |  |
|  |  | TGACG-motif | TGACG | cis-acting regulatory element involved in the MeJA-responsiveness | 1 | 1 |  |  |  |  |  |  |  |
|  |  | CGTCA-motif | CGTCA | cis-acting regulatory element involved in the MeJA-responsiveness | 1 | 1 |  |  |  |  |  |  |  |
|  |  | TATC-box | TATCCCA | cis-acting element involved in gibberellin-responsiveness |  | 1 |  |  |  |  |  |  |  |
|  |  | MBSI | aaaAaaC(G/C)GTTA | MYB binding site involved in flavonoid biosynthetic genes regulation |  |  |  |  | 1 | 1 |  |  |  |
| Others | 17 | CAAT-box | CAAAT | common cis-acting element in promoter and enhancer regions | 1 | 1 | 1 |  | 1 | 1 | 1 |  |  |
|  |  |  | CCAAT | common cis-acting element in promoter and enhancer regions |  |  |  |  |  |  |  |  |  |
|  |  |  | CAAT | common cis-acting element in promoter and enhancer regions |  |  |  |  |  |  |  |  |  |
|  |  |  | TGCCAAC | common cis-acting element in promoter and enhancer regions |  |  |  |  |  |  |  |  |  |
|  |  | TATA-box | ccTATAAAaa | core promoter element around -30 of transcription start | 1 | 1 | 1 |  | 1 | 1 | 1 |  |  |
|  |  |  | TATA | core promoter element around -30 of transcription start |  |  |  |  |  |  |  |  |  |
|  |  |  | TATAAA | core promoter element around -30 of transcription start |  |  |  |  |  |  |  |  |  |
|  |  |  | TATACA | core promoter element around -30 of transcription start |  |  |  |  |  |  |  |  |  |
|  |  |  | TACAAAA | core promoter element around -30 of transcription start |  |  |  |  |  |  |  |  |  |
|  |  |  | TATAAAA | core promoter element around -30 of transcription start |  |  |  |  |  |  |  |  |  |
|  |  |  | TATAA | core promoter element around -30 of transcription start |  |  |  |  |  |  |  |  |  |
|  |  |  | TCTTAC | core promoter element around -30 of transcription start |  |  |  |  |  |  |  |  |  |
|  |  |  | TACATAAA | core promoter element around -30 of transcription start |  |  |  |  |  |  |  |  |  |
|  |  |  | TATAAATA | core promoter element around -30 of transcription start |  |  |  |  |  |  |  |  |  |
|  |  |  | ATATAA | core promoter element around -30 of transcription start |  |  |  |  |  |  |  |  |  |
|  |  |  | ATTATA | core promoter element around -30 of transcription start |  |  |  |  |  |  |  |  |  |
|  |  |  | TATAAAT | core promoter element around -30 of transcription start |  |  |  |  |  |  |  |  |  |
|  |  |  | ATATAT | core promoter element around -30 of transcription start |  |  |  |  |  |  |  |  |  |
|  |  |  | taTATAAAtc | core promoter element around -30 of transcription start |  |  |  |  |  |  |  |  |  |
|  |  | A-box | CCGTCC | cis-acting regulatory element |  |  |  | 1 |  |  |  |  |  |
|  |  | CCAAT-box | CAACGG | MYBHv1 binding site |  |  | 1 |  |  |  |  |  |  |
|  |  | AT-rich element | ATAGAAATCAA | binding site of AT-rich DNA binding protein (ATBP-1) |  | 1 |  |  |  |  |  |  |  |
